# Omitting radiotherapy is safe in breast cancer patients ≥ 70 years old after breast-conserving surgery without axillary lymph node operation

**DOI:** 10.1038/s41598-020-76663-5

**Published:** 2020-11-10

**Authors:** Ying Zhong, Yali Xu, Yidong Zhou, Feng Mao, Yan Lin, Jinghong Guan, Songjie Shen, Bo Pan, Changjun Wang, Li Peng, Xin Huang, Xuefei Wang, Qiang Sun

**Affiliations:** Department of Breast Disease, Peking Union Medical College Hospital, No.1 Shuaifuyuan, Wangfujing, Beijing, 100730 China

**Keywords:** Breast cancer, Risk factors

## Abstract

To verify whether omitting radiotherapy from breast cancer treatment for patients ≥ 70 years old following breast-conserving surgery (BCS) without axillary lymph node dissection is safe. Previous studies have shown that omitting breast radiotherapy after BCS and axillary lymph node dissection is safe for elderly breast cancer patients. We aimed to evaluate the safety of BCS without axillary surgery or breast radiotherapy (BCSNR) in elderly patients with breast cancer and clinically negative axillary lymph nodes. We performed a retrospective analysis of 481 patients with breast cancer, aged ≥ 70 years, between 2010 and 2016. Of these, 302 patients underwent BCSNR and 179 underwent other, larger scope operations. Local recurrence rate, ipsilateral breast tumor recurrence (IBTR) rate, distant metastasis rate, breast-related death, disease-free survival (DFS), and overall survival (OS) were compared between the two groups. After a median follow-up of 60 months, no significant differences in local recurrence, distant metastasis rate, breast-related death, and DFS were noted. The OS was similar (P = 0.56) between the BCSNR group (91.7%) and other operations group (93.0%). The IBTR rate was considered low in both groups, however resulted greater (P = 0.005) in the BCSNR group (5.3%) than in other operations group (1.6%). BCSNR did not affect the survival of elderly patients with breast cancer with clinically negative axillary lymph nodes. IBTR was infrequent in both groups; however, there was a significant difference between the two groups. BCSNR is a feasible treatment modality for patients with breast cancer ≥ 70 years old with clinically negative axillary lymph nodes.

## Introduction

In women, breast cancer incidence rates far exceed other cancers worldwide, accounting for nearly 1 in 4 cancer cases^1^. Furthermore, breast cancer is the leading cause of cancer death among women^1^. The incidence of breast cancer increases with age^2,3^; in China, the second most frequent age group diagnosed with breast cancer is 70–74 years^4^. At the time of diagnosis, more than 35% of patients are > 65 years old^5^. Elderly patients with breast cancer form a large, heterogeneous group, and many patients have concomitant diseases and senility^6^. Studies have found that the overall survival (OS) rate of patients with complications after surgery is lower than that of patients without complications^7^. Furthermore, postoperative complications in elderly patients with breast cancer also increase with age.

Breast cancer is classified into five intrinsic subtypes by gene expression microarray: luminal A, luminal B, human epidermal growth factor receptor 2 (HER2)-enriched, basal-like, and normal-like^8^. Favorable subtypes (luminal tumors) increase with age, whereas subtypes with poor outcomes (basal-like tumors) decrease with age^8^. The prognosis of elderly patients with breast cancer is good and most of them die from non-breast cancer diseases^9^. The mortality of patients with breast cancer older than 50 years gradually decreases with age^10^; therefore, some studies recommend that surgical scope or the use of adjuvant treatments for elderly patients with breast cancer should be reduced. In elderly patients with breast cancer and clinically node-negative disease do not perform axillary clearance yields similar efficacy than perform axillary clearance^11^. Although radiotherapy is a standard recommendation in patients with breast cancer^12^, in low- and medium-risk patients, omitting radiotherapy did not cause an increase in mortality^13^. In the CALGB 9343 trial^14^, low-risk and elderly patients with breast cancer who underwent systemic therapy were randomly divided into radiotherapy and non-radiotherapy groups. The disease-free survival (DFS) and OS were not significantly different between the two groups.

Therefore, we aimed to evaluate whether the survival of elderly patients with breast cancer is affected when solely local enlarged tumors are excised without axillary lymph node dissection nor radiotherapy.

## Materials and methods

The Peking Union Medical College Hospital (PUMCH) database includes the data of patients with breast cancer who have underwent treatment in this hospital since 1975. The data includes age, operation methods, radiotherapy, chemotherapy, endocrine therapy, concomitant diseases, recurrence, metastasis, and other information. Any hard copies of patient records were electronically filed by scanning. The necessary data was extracted and inputted into a new database^15^.

Since 1975, more than 2000 patients with breast cancer over 70 years old had operations and other treatments at our hospital. This study included 481 patients with breast cancer aged over 70 years. All patients underwent surgery between 2010 and 2016 and underwent other treatments and clinical visits at PUMCH. All patients provided written informed consent.

The study was approved by the Institutional Review Board of Peking Union Medical College Hospital (S-K971). All study participants provided informed consent and all the patients’ data were anonymous. The present research was performed in accordance with relevant regulations.

### Patient data

Among 481 patients who underwent operations at our hospital, 302 underwent breast-conserving surgery without axillary lymph node dissection, sentinel lymph node biopsy, or radiotherapy (BCSNR). Patients with a life expectancy of less than 3 months were not included in our study. All patients were informed of the surgical options, including BCSNR, mastectomy plus axillary lymph nodes dissection, mastectomy plus sentinel lymph node biopsy, breast-conserving surgery plus axillary lymph nodes dissection, and breast-conserving surgery plus sentinel lymph node biopsy. Surgeons communicated the operation risks, advantages, and disadvantages to the patients before treatment. The operation method chosen depended on patients’ preferences and doctors’ opinions.

All patients had a preoperative clinical evaluation that included physical examination (PE), ultrasonography, and mammography. If the patients had underwent BCSNR, PE for axillary had been negative with no suspicious calcification on the mammograph or other suspicious tumors in multiple quadrants on the ultrasound. Patients with clinically suspected lymph nodes underwent axillary lymph nodes dissection or sentinel lymph node biopsy. Axillary dissection was performed in patients with enlarged suspicious lymph nodes by PE. Sentinel lymph node biopsy was performed in patients with no enlarged lymph nodes by PE but with suspicious lymph nodes in ultrasonography or mammography. The clinical and radiological free lymph nodes include clear boundary between cortex and medulla, no suspicious blood flow, no abnormal morphology, no cortical thickening and without enlarged lymph nodes by PE.

### Follow-up

The median follow-up period was 60 months from the date of operation. The shortest follow-up period was 5 months and the longest was 111 months.

### Tumor characteristics

Pathological information from all patients was obtained from the Department of Pathology at PUMCH. Estrogen receptor (ER)- and progestogen receptor (PR)-positive disease were defined by immunohistochemical (IHC) staining of > 1% of cells. HER2-positive was defined by HER2 protein expression IHC 3+ positive and if HER2 was 2+ positive on IHC, we performed immunofluorescence hybridization (FISH) for HER2. FISH was positive if the average HER2 gene copy number ≥ 6.0 signals/cell or HER2/CEP17 ratio ≥ 2.0^16^. T stage was categorized as T1 (< 5 cm), T2 (> 2 cm, ≤ 5 cm), T3 (> 5 cm) or T4 (tumor of any size with direct extension to the chest wall or to the skin). Pathological axillary lymph node stage was categorized as N0 (0 positive axillary lymph nodes), N1 (metastases to 1–3 axillary lymph nodes), N2 (metastases to 4–9 axillary lymph nodes), N3 (metastases to ≥ 10 axillary lymph nodes). Histology type was determined according to the World Health Organization classification^17^.

### Outcome variables

Regional recurrence included local breast recurrence, chest wall recurrence, or axillary lymph node recurrence. Breast cancer metastasis included bone, liver, lung, and brain metastases or distant skin metastasis. DFS was defined as survival without recurrence or metastasis. OS was defined as survival without death. Breast cancer-related death was defined as death caused by the recurrence or metastasis of breast cancer. Non-breast cancer-related death was defined as death caused by a concomitant non-breast cancer disease.

### Statistical analysis

All statistical analyses were performed with SPSS 22.0 software. All statistical tests were two-sided, and significance was defined as *P* < 0.05. Baseline characteristics of patients were analyzed by χ^2^ and Mann–Whitney U-tests. OS and DFS were calculated using Kaplan–Meier analysis. The associations between recurrence, metastasis, and operation method were evaluated by Mann–Whitney U-tests. Tests for interaction between age group, T stage, histology type, HR status, molecular type, and operation method and DFS and OS were analyzed by univariate Cox regression analysis and a multivariate Cox regression model.

## Results

The median age of 481 patients was 76 (range 70–91) years. Of 481 patients, 286 (38.7%) patients were 70–74 years old, 171 (35.6%) patients were 75–79 years old and 124 (25.8%) patients were > 80 years old. Regarding operations, 302 (62.8%) patients underwent BCSNR, 148 (30.8%) underwent mastectomy plus axillary lymph nodes dissection, 18 (3.7%) underwent breast-conserving surgery plus axillary lymph nodes dissection, seven (1.5%) underwent mastectomy plus sentinel lymph node biopsy, and six (1.2%) underwent breast-conserving surgery plus sentinel lymph node biopsy. Thirty patients underwent chemotherapy with capecitabine. Regarding HR status, 382 (79.4%) patients had HR-positive disease and underwent aromatase inhibitor treatment. Regarding breast cancer subtype, 176 (36.6%) patients had the luminal A subtype, 208 (43.2%) had the luminal B subtype, 37 (7.7%) had the HER2-enriched subtype, and 60 (12.5%) had triple-negative breast cancer. Most patients had T1 and T2 stage tumors; 284 (59.0%) patients had T1 stage tumors and 182 (37.8%) patients had T2 tumors. Regarding histological grade, 105 (21.8%) patients had grade 1 and 231 (48.0%) had grade 2 (Table 1).

**Table 1:** Patients and clinical characteristics.

	Total	BCSNR	Mastectomy and axillary lymph node dissection	breast conserving surgery and axillary lymph node dissection	Mastectomy and sentinel lymph node biopsy	breast conserving surgery and sentinel lymph node biopsy
Age	N = 481	302	148	18	7	6
70–74	186 (38.7%)	94 (31.1%)	79 (53.4%)	6 (33.3%)	5 (71.4%)	2 (33.3%)
75–59	171 (35.6%)	106 (35.1%)	54 (36.5%)	8 (44.4%)	2 (28.6%)	1 (16.7%)
80+	124 (25.8%)	102(33.8%)	15 (10.1%)	4 (22.2%)	0 (0.0%)	3 (50.0%)
**T stage**						
0	1 (0.2%)	0 (0.0%)	1 (0.7%)	0 (0.0%)	0 (0.0%)	0
1	284 (59.0%)	193 (63.9%)	73 (49.3%)	17 (94.4%)	1 (14.3%)	1 (16.7%)
2	182 (37.8%)	105 (34.8%)	66 (44.6%)	1 (5.6%)	6 (85.7%)	4 (66.7%)
3	13 (2.7%)	4 (1.3%)	8 (5.4%)	0 (0.0%)	0 (0.0%)	1 (16.7%)
4	1 (0.2%)	0 (0.0%)	1 (0.7%)	0 (0.0%)	0 (0.0%)	0
**Histological grade**						
Un-known	71 (14.8%)	45 (14.9%)	19 (12.8%)	4 (22.2%)	2 (28.6%)	1 (16.7%)
1	105 (21.8%)	53 (17.5%)	46 (31.1%)	4 (22.2%)	1 (14.3%)	1 (16.7%)
2	231 (48.0%)	144 (47.7%)	72 (48.6%)	9 (50.0%)	2 (28.6%)	4 (66.7%)
3	74 (15.4%)	60 (19.9%)	11 (7.4%)	1 (5.6%)	2 (28.6%)	0 (0.0%)
**HR**						
−	99 (20.6%)	52 (17.2%)	42 (28.4%)	3 (16.7%)	1 (14.3%)	1 (16.7%)
+	382(79.4%)	250 (82.8%)	106 (71.6%)	15 (83.3%)	6 (85.7%)	5 (83.3%)
**Molecular type**						
Luminal A	176 (36.6%)	125 (41.4%)	36 (24.3%)	8 (44.4%)	6 (85.7%)	1 (16.7%)
Luminal B	208 (43.2%)	125 (41.4%)	71 (48.0%)	8 (44.4%)	0 (0.0%)	4 (66.7%)
HER2	37 (7.7%)	15 (5.0%)	21 (14.2%)	0 (0.0%)	1 (14.3%)	0 (0.0%)
TNBC	60 (12.5%)	37 (12.3%)	20 (13.5%)	2 (11.1%)	0 (0.0%)	1 (16.7%)
**LN stage**						
0			78 (52.7%)	14 (77.8%)	7 (100.0%)	1 (16.7%)
1			27 (18.2%)	4 (22.2%)	0 (0.0%)	2 (33.3%)
2			26 (17.6%)	0 (0.0%)	0 (0.0%)	2 (33.3%)
3			17 (11.5%)	0 (0.0%)	0 (0.0%)	1 (16.7%)

### Recurrence

During follow-up, 31 regional recurrences, of which 17 were ipsilateral breast tumor recurrences (IBTR), and 13 distant recurrences were observed. In patients who underwent BCSNR, 21 regional recurrences, including 16 IBTR and seven distant recurrences, were observed. In patients who underwent other operations, 10 regional recurrences, including one IBTR and six distant recurrences were observed. The IBTR rates between the two groups were significantly different, which was higher in BCSNR group (*P* = 0.005). However, regional and distant recurrences were not significantly different between the two groups (Table 2).

**Table 2:** Regional recurrence, distant recurrence and IBTR analyzed by operation methods.

	Total	BCSNR	Other operations	P value
Regional recurrence	31 (6.4%)	21 (7.0%)	10 (5.6%)	0.70
Distant recurrence	13 (2.7%)	7 (2.3%)	6 (3.4%)	0.57
IBTR	17 (3.5%)	16 (5.3%)	1 (0.6%)	0.005

### Survival

There was a total of 48 deaths among the patients. Of these, 11(2.3%) were breast cancer-related and 37 (7.7%) were non-breast cancer-related. In the BCSNR group, there were 6 (2.0%) breast cancer-related deaths and 23 (7.6%) non-breast cancer-related deaths. In the other operations group, there were 5 (2.8%) breast cancer-related deaths and 14 (7.8%) non-breast cancer-related deaths (Table 3). The breast cancer-related and non-breast cancer-related deaths were not significantly different between the two groups.

**Table 3:** Mortality in relation to different operation methods.

	Total	BCSNR	Other operations	P value
Breast cancer related death	11 (2.3%)	6 (2.0%)	5 (2.8%)	1.00
Non-breast cancer related death	37 (7.7%)	23 (7.6%)	14 (7.8%)	1.00
Total death	48 (10.0%)	29 (9.6%)	19 (10.6%)	0.75

**Figure 1:**
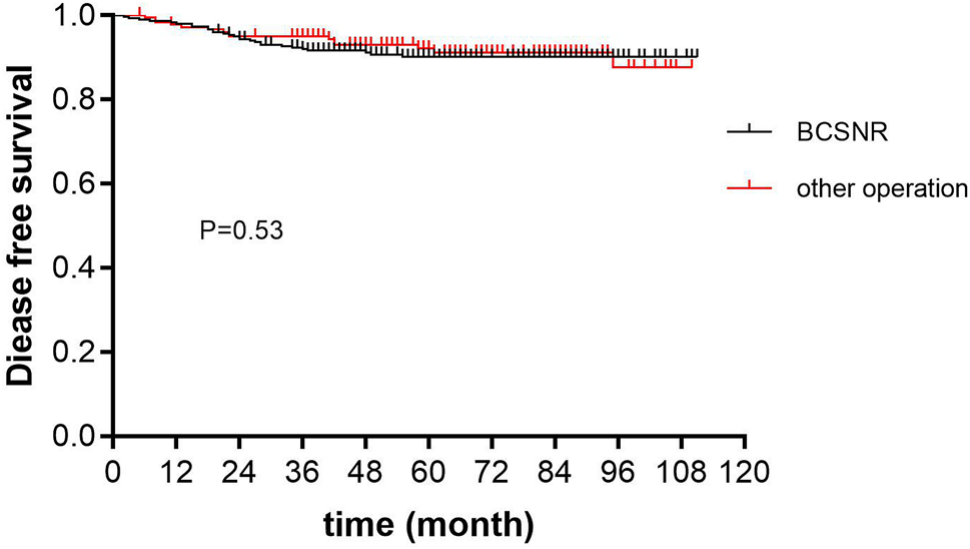
Five-year disease free survival.

**Figure 2:**
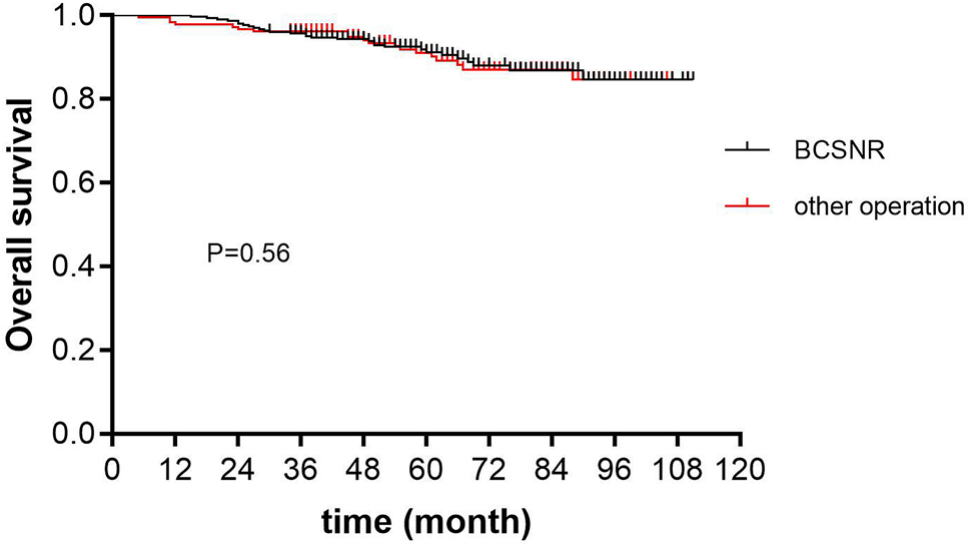
Five-year overall survival.

Kaplan–Meier analysis showed that the 5-year DFS was 91.6% and 92.9% in the BCSNR and other operations groups, respectively, and was not significantly different (*P* = 0.53; Fig. 1). Furthermore, the 5-year OS was 91.7% and 93.0% in the BCSNR and other operations groups, respectively, and was not significantly different (*P* = 0.56; Fig. 2).

Univariate and multivariate Cox regression analyses showed that patients who underwent BCSNR had a similar DFS to that of patients who underwent other operations. According to the multivariate analysis, using patients who underwent BCSNR as a reference, the hazard ratio for patients who underwent other operations was 0.76 [95% confidence interval (CI) (0.39–1.47); Table 4].

**Table 4:** Univariate and multivariable Cox analyses of age, T stage, histology type, HR status, molecular type and operation method on disease free survival.

Clinical character	Univariable analysis			Multivariate analysis		
	HR^b^	95% CI	P value	HR^b^	95% CI	P value
**Age group**						
70–74	1.00	Ref		1.00	Ref	
75–79	0.93	0.43–2.01	0.86	1.02	0.46–2.26	0.96
80+	1.03	0.48–2.23	0.94	1.32	0.60–2.91	0.50
T stage^a^	1.16	0.70–1.92	0.575	0.98	0.56–1.72	0.94
Histological type^a^	1.02	0.74–1.41	0.89	1.29	0.87–1.91	0.21
HR^c^	0.26	0.14–0.47	0.00	2.00	0.41–9.77	0.39
Molecular type^a^	1.65	1.37–2.00	0.00	2.15	1.28–3.59	0.004
Operation method	0.87	0.46–1.63	0.66	0.76	0.39–1.47	0.41

^a^These clinical features are considered continuous variables, giving the increased hazard ratio per unit.

^b^Hazard ratio.

^c^Hormone receptor.

Univariate and multivariate Cox regression analyses showed that patients who underwent BCSNR had a similar OS to that of patients who underwent other operations. According to the multivariate analysis, using patients who underwent BCSNR as a reference, the hazard ratio for patients who underwent other operations was 1.16 [95% CI (0.62–2.17); Table 5].

**Table 5:** Univariate and multivariable Cox analyses of age, T stage, histology type, HR status, molecular type and operation method on overall survival.

Clinical character	Univariable analysis			Multivariate analysis		
	HR^b^	95% CI	P value	HR^b^	95% CI	P value
**Age group**						
70–74	1.00	Ref				
75–79	0.46	0.23–0.92	0.028	0.45	0.22–0.95	0.035
80+	0.52	0.26–1.02	0.058	0.51	0.25–1.04	0.064
T stage^a^	1.77	1.15–2.71	0.009	1.64	1.03–2.62	0.039
Histological type^a^	1.12	0.82–1.53	0.465	1.40	0.97–2.02	0.073
HR^c^	0.35	0.20–0.61	0.000	0.34	0.09–1.24	0.10
Molecular type^a^	1.53	1.19–1.97	0.001	1.04	0.60–1.82	0.89
Operation method	1.02	0.57–1.83	0.936	1.16	0.62–2.17	0.66

^a^These clinical features are considered continuous variables, giving the increased hazard ratio per unit.

^b^Hazard ratio.

^c^Hormone receptor.

Associations between DFS and OS and age, T stage, histology type, HR status, and molecular subtype were evaluated. Univariate Cox regression analysis demonstrated that HR status and molecular subtype was significantly associated with DFS. Multivariate analysis demonstrated that molecular subtype was significantly associated with DFS (*P* = 0.004; Table 4). Univariate Cox regression analysis demonstrated that the T stage, HR status, and molecular subtype were significantly associated with OS. Multivariate analysis demonstrated that age and T stage were significantly associated with OS (*P* = 0.035 and *P* = 0.039 respectively; Table 5).

## Discussion

With the aging population, the incidence of breast cancer in elderly women is increasing worldwide^18^. In elderly patients with breast cancer, there is a greater incidence of HR-positive and HER2-negative patients, with a lower incidence of triple-negative breast cancer^19^. As age increases, breast cancer-related mortality decreases, whereas non-breast cancer-related mortality increases^14^. The incidence of postoperative complications also increases with age in these patients^7^. A study on patients with breast cancer ≥ 70 years of age randomly divided patients into two groups, tamoxifen vs. tamoxifen + mastectomy^20^. There was no significant difference in breast cancer-related deaths after the 10-year follow-up. However, local control in the tamoxifen group was poor at 1.9% compared to 43.0% in the tamoxifen + mastectomy group. Therefore, local operation cannot be omitted for elderly patients with breast cancer. However, some studies suggest that the operation scope and treatment measures for breast cancer can be reduced in elderly patients^21^.

Martelli et al.^21^ assessed the long-term safety of forgoing axillary clearance in elderly patients with breast cancer. After a median follow-up of 15 years, there was no significant difference in breast cancer-related mortality between the axillary clearance and no axillary clearance groups. The crude cumulative 15-year incidence of axillary disease in the no axillary clearance group was low at 5.8% for all patients and 3.7% for the pT1 (tumor size < 2 cm) subgroup. The IBCSG Trial 10–93^11^ also investigated primary surgery plus axillary clearance versus surgery without axillary clearance and found that omitting axillary clearance for elderly women with clinically node-negative disease yielded similar efficacy with better early quality of life. Both studies compared the survival benefits of patients with axillary dissection to those without axillary dissection, however in the former study, 229 of 691 patients underwent breast irradiation, and in the latter study, more than half of the patients underwent breast-conserving surgery with radiotherapy. It has also been reported that sentinel lymph node biopsy can be omitted in elderly patients^22^. In this study, low-risk patients or those with T1 tumors were recommended not to receive sentinel lymph node biopsy. Additionally, Several studies have also found that the local recurrence rate is relatively low for elderly patients with breast cancer treated with breast-conserving surgery without radiotherapy^23,24^. Therefore, we aimed to evaluate whether breast-conserving surgery without axillary lymph nodes dissection, sentinel lymph node biopsy, nor radiotherapy affects the survival outcomes of elderly patients.

Our study showed that there was no significant difference in DFS between the BCSNR and other operations groups. Similarly, there was no significant difference between the two groups in OS. Our results are similar to those of two previous studies^11,21^ reporting axillary lymph node dissection did not affect the survival of elderly patients with breast cancer. In Trial 10–93, results for surgery plus axillary clearance and surgery without axillary clearance yielded similar OS (75% vs 73%; *P* = 0.77) at a median follow-up of 6.6 years^11^. In Martelli’s study, the 15-year crude cumulative incidence of breast cancer death was 14.0% in the no axillary dissection group and 13.6% in the axillary dissection group (*P* = 0.657)^21^. However, in our study, 302 patients who underwent breast-conserving surgery did not receive radiotherapy. The IBTR rate in patients who underwent BCSNR was significantly higher than in patients who underwent other operations. This result is similar to those of the PRIME II study^25^. In that study, 658 women who underwent breast-conserving surgery were randomly assigned to receive whole-breast irradiation and 668 did not receive whole-breast irradiation. After 5 years of follow-up, the IBTR rate was 1.3% in women who underwent radiotherapy and 4.1% in those assigned no radiotherapy (*P* = 0.0002). The authors of that study suggested that the IBTR rate was low enough for the omission of radiotherapy to be considered for some patients. The CALGB 9343 trial reached similar conclusions^14^. In that clinical trial, 636 patients (age ≥ 70 years) who underwent lumpectomies were randomly assigned to receive tamoxifen plus radiation therapy or tamoxifen alone. After 10 years of follow-up, 98% of the patients receiving tamoxifen plus radiation were free from local and regional recurrences compared to 90% of those receiving tamoxifen alone (95% CI 85–93%).

Furthermore, we found that HR status, molecular subtype, age, and T stage were related to the prognosis of patients. HR status was significantly associated with DFS by univariate analysis, and the prognoses of patients with HR-positive disease were better than those with HR-negative disease. Molecular subtype was significantly correlated with DFS by multivariable analysis, and the prognoses of the luminal type subgroups were significantly better than those of the poor molecular type subgroups. Similar to the findings of Syed et al.^9^, the higher the HR-positive rate in elderly patients, the better the prognosis was. T stage was significantly correlated with OS by multivariate analysis, and the prognoses of patients with small tumor size were significantly better than those of patients with large tumor size. This result is similar to those reported by Hongchao et al.^26^, in which the risk of breast cancer-related death was dependent on T status, and patients with early-T stage disease had better prognoses than those with late-T stage disease. We found that the risk of death in patients aged 75–79 years was significantly lower than that in patients aged 70–74 years, in line with previous reports^10^; as patient age increased, breast cancer-related mortality decreased. Univariate Cox regression showed that age had no significant correlation with DFS, but it was related to OS, indicating that the prognosis of elderly breast cancer patients may be closely related to their own basic diseases and physical conditions, but not to breast cancer disease itself and the operation method we choose. There was no significant difference in OS between patients over 80 years old and those 70–74 years old, which may be due to the confounding factor of non-breast cancer-related death.

In our study, we evaluated the recurrence, metastasis, and survival results of elderly patients with breast cancer who underwent BCSNR. We further compared these patients with those who underwent surgery involving other methods, including breast-conserving surgery plus axillary lymph nodes dissection or sentinel lymph node biopsy and mastectomy plus axillary lymph nodes dissection or sentinel lymph node biopsy. We have demonstrated that BCSNR is safe and reliable for elderly patients with breast cancer. Future studies should include more patients and better match the clinical characteristics of patients who receive BCSNR with those who have other operations. Additionally, prolonged follow-up periods could further support the results of future studies.

In conclusion, we found that elderly patients with breast cancer treated with BCSNR had similar DFS and OS to patients who underwent larger scope operations. Therefore, BCSNR is a feasible treatment modality for patients with breast cancer ≥ 70 years old with clinically negative axillary lymph nodes.
